# Making Headway: The Roles of Hox Genes and Neural Crest Cells in Craniofacial Development

**DOI:** 10.1100/tsw.2003.11

**Published:** 2003-04-14

**Authors:** Paul A. Trainor

**Affiliations:** Stowers Institute for Medical Research, 1000 East 50th Street, Kansas City, MO 64110, USA

**Keywords:** Hox genes, neural crest cells, craniofacial development, mouse, chick, embryo, branchial arch, pharyngeal arch, plasticity, neural tube, rhombomere, segmentation, mesoderm, ectoderm, endoderm, evolution

## Abstract

Craniofacial development is an extraordinarily complex process requiring the orchestrated integration of multiple specialized tissues such as the surface ectoderm, neural crest, mesoderm, and pharyngeal endoderm in order to generate the central and peripheral nervous systems, axial skeleton, musculature, and connective tissues of the head and face. How do the characteristic facial structures develop in the appropriate locations with their correct shapes and sizes, given the widely divergent patterns of cell movements that occur during head development? The patterning information could depend upon localized interactions between the epithelial and mesenchymal tissues or alternatively, the developmental program for the characteristic facial structures could be intrinsic to each individual tissue precursor. Understanding the mechanisms that control vertebrate head development is an important issue since craniofacial anomalies constitute nearly one third of all human congenital defects. This review discusses recent advances in our understanding of neural crest cell patterning and the dynamic nature of the tissue interactions that are required for normal craniofacial development.

## ORGANISATION OF THE HINDBRAIN AND ITS INFLUENCE ON CRANIOFACIAL DEVELOPMENT

The vertebrate hindbrain is one key source of patterning information and it exerts a profound influence on craniofacial development. During early vertebrate embryo development, the hindbrain becomes transiently subdivided into seven contiguous cell lineage restricted compartments called rhombomeres (r) (Fig. 1)[1]. Each rhombomere adopts a distinct set of molecular and cellular properties including restrictions in cell mixing and gives rise to unique regions of the mature adult brain[2,3,4,5,6,7,8,9]. The segmental organization of the hindbrain presages the establishment of an anatomical and functional registration between individual rhombomeres, cranial ganglia, branchiomotor nerves, and the migration pathways of cranial neural crest cells into the branchial arches[10,11,12].

In chick embryos, the formation and disposition of motor neurons conforms to a two-segment periodicity. The trigeminal (Vth), facio-acoustic (VIIth), and glossopharyngeal (IXth) motor nerves innervate the first three branchial arches, respectively. Although each nerve is derived from neurons born in multiple rhombomeres, the axons leave the hindbrain through exit points contained only within the even-numbered rhombomeres (Fig. 1)[10,11]. Similar to its influence on cranial nerve patterning, the rhombomeric organization of the hindbrain also influences the pathways of cranial neural crest cell migration. The hindbrain gives rise to the majority of cranial neural crest cells which migrate ventrolaterally in three discrete segmental streams adjacent to the even-numbered rhombomeres. The streams of cranial neural crest cells follow a subectodermal route over the surface of the cranial mesoderm and populate the first, second, and third branchial arches, respectively, in keeping with their craniocaudal origins (Fig. 1)[13,14,15,16]. Although almost the entire cranial neural tube generates neural crest cells, it appears that substantially less neural crest cells delaminate from the odd rhombomeres compared with the even-numbered rhombomeres. In chick embryos, this phenomenon is believed to be associated with odd rhombomere specific apoptosis of neural crest precursors[17,18,19]. Rather than delaminating and migrating laterally like the rest of the hindbrain neural crest, lineage tracing and time-lapse analyses in numerous vertebrates, show that r3- and r5-derived neural crest cells migrate both rostrally and caudally joining the even-numbered streams as they contribute to the proximal most regions of the branchial arches[12,14,15,16,20,21,22,23]. Gene mutations—which disrupt the segmental patterning of the hindbrain—result in fusions of the cranial ganglia, branchiomotor defects, and abnormal patterns of cranial neural crest cell migration[24,25,26,27,28,29,30,31,32,33]. Therefore, the segmental organization of the hindbrain is essential for establishing the foundations of head development and is a conserved strategy used by vertebrates to ensure proper craniofacial morphogenesis.

## MECHANISMS FOR SEGMENTATION OF THE HINDBRAIN

The establishment of segment identity and the maintenance of organized patterns of gene expression during hindbrain development requires the restriction of cell intermingling between adjacent segments. Cells with similar adhesive properties such as those from odd or even rhombomeres display a preferential association[34,35]. Consequently, it was hypothesized that segregating the distinct cell populations was facilitated by a hierarchy of cell adhesion interactions[36]. The appearance of restricted domains of gene expression in the hindbrain coincides with rhombomere partitioning and the adoption of neural character (Fig. 1). Numerous genes including transcription factors, signalling molecules, membrane and nuclear receptors are all dynamically expressed in segmentally restricted patterns during hindbrain development (reviewed in [37,38,39]). Some genes are expressed in single rhombomeres, a few are confined to pairs of rhombomeres, while others are found only at rhombomere boundaries. Regulatory and mutational analyses in mice and other vertebrate species have helped to uncover the molecular and cellular mechanisms involved in subdividing the hindbrain into rhombomeres.

Restrictions in cell mixing are crucial for establishing the unique segmental identities of each rhombomere and the *Eph* family of receptor tyrosine kinases and their ligands, the *ephrins*, play key roles in this process[40]. The *Eph* receptors and their ephrin ligands are expressed in mutually exclusive domains in the hindbrain such that each receptor and its associated ligand is expressed in an alternate, nonoverlapping pattern in adjacent rhombomeres (Fig. 1)[40,41,42,43]. For example, during the 8.0–10.5 days postcoitum (dpc) period of mouse embryonic development, *EphB* receptors are expressed in odd-numbered rhombomeres and their corresponding *ephrinB* ligands are expressed in the adjacent even-numbered rhombomeres. In addition to the *EphB* receptors, the receptors *EphA2* and *EphA4* are also segmentally expressed in hindbrain rhombomeres (Fig. 1). The mosaic activation of *Eph* receptors or their *ephrin* ligands in the hindbrain leads to cell sorting at the boundaries of odd and even rhombomeres, respectively. This suggests that bidirectional signalling at rhombomere boundaries restricts cell intermingling[44,45]. Therefore bipartite *Eph/ephrin* contact-mediated repulsion maintains inter-rhombomeric boundaries which are essential for establishing the hindbrain compartments as units of cell lineage restriction.

## SPECIFYING SEGMENTAL IDENTITY IN THE HINDBRAIN

Genetic analyses in several vertebrate species have shown that *Hox* genes are essential for specifying segmental identity in the developing hindbrain. The *Hox* gene family comprises a set of developmentally regulated transcription factors that are characterized by a conserved DNA binding motif called the homeobox. *Hox* genes are organized into a single chromosomal cluster in invertebrates, in contrast to higher vertebrates such as the mouse which have 39 *Hox* genes, organized into four distinct chromosomal clusters (*Hoxa-Hoxd*) located on different chromosomes[46]. This arrangement arose during evolution from a single ancestral homeobox as a consequence of duplication and divergence[47]. The most striking feature of the organization of *Hox* gene family members is the spatial and temporal colinearity, which confers positional information along the body axis[46,48,49,50]. Genes located nearer to the 3’ end of the cluster are expressed earlier and more anteriorly during development than those located nearer the 5’ end, such that *Hox* genes exhibit nested domains of expression along the anterior-posterior (A-P) axis of the neural tube. Only the 3’ members of each complex (paralogue groups 1-4) are expressed in the vertebrate hindbrain and these *Hox* genes exhibit dynamic patterns of expression during hindbrain development (Fig. 1)[51].

*Hoxa1* and *Hoxb1* are initially expressed in the neural tube up to the presumptive r3/4 boundaries, but as *Hoxa1* expression regresses, *Hoxb1* expression becomes confined to r4 by 8.5dpc during mouse embryo development[52,53,54]. *Hoxa2* expression is initiated in the neural epithelium at 7.5dpc and by 8.5dpc it is uniformly expressed up to the r1/r2 boundary[51]. Shortly thereafter *Hoxa2* becomes expressed at significantly higher levels in r3 and r5[55]. In contrast, *Hoxb2* is expressed up to the r2/r3 boundary around the same time, but its expression levels are subsequently upregulated in r3, r4, and r5[56,57]. Group 3 (*Hoxa3, Hoxb3, Hoxd3*) genes are expressed anteriorly up to the r4/r5 boundary, with *Hoxa3* exhibiting upregulation in r5 and r6 and *Hoxb3* being similarly upregulated in r5[58,59]. In contrast, *Hoxd3* is expressed at lower levels in r5 than more posterior regions. Group 4 genes (*Hoxa4, Hoxb4, Hoxd4,* and *Hoxc4*) are generally expressed up to the r6/r7 boundary by 9.5dpc, however their precise anterior limits vary slightly (Fig. 1)[60,61].

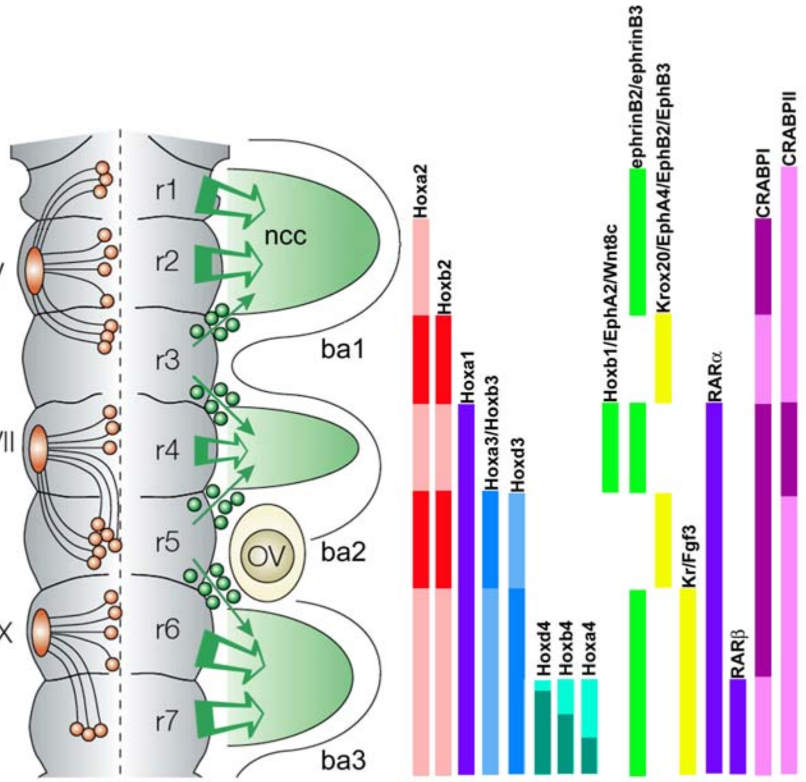

**FIGURE 1**. Segmentation of the hindbrain, motor nerves, and pathways of neural crest cell (ncc) migration. The vertebrate hindbrain becomes subdivided into 7 transient rhombomeres (r) which presages the segmental organization of the branchiomotor nerves, III (trigeminal), V (facial), and VII (glossopharyngeal) and their innervation of the first (ba1), second (ba2), and third (ba3) branchial arches, respectively. Although the cell bodies (small red circles) that make up each branchiomotor nerve are derived from multiple rhombomeres, their axons project and leave the neural tube only from exit points contained within the even numbered rhombomeres (large red circles). Neural crest cells derived from the hindbrain (large green arrows) migrate ventrolaterally in three segmental streams adjacent to the even-numbered rhombomeres and into the branchial arches. Neural crest cells derived from the odd-numbered rhombomeres do not migrate laterally, rather they migrate anteriorly or posteriorly to join the even-numbered neural crest streams. The hindbrain segmentation process which establishes each rhombomere as a unit of cell lineage and the subsequent imposition of cell fate identity corresponds with the activation of expression, of genes from multiple families, such as *Hox*, *Eph/ephrin*, *RAR* and *CRABP* families. The patterns of gene expression are extremely dynamic and only the primary pattern for each gene between 8.5–10.5dpc is shown here. The only exception is *Hoxa1* which is expressed up to the presumptive r3/4 boundary at 7.5dpc but is subsequently switched off before rhombomere formation. OV, otic vesicle.

## GENETIC CONTROL OF *HOX* GENE EXPRESSION AND HINDBRAIN PATTERNING

*Hox* genes are an integral part of the process specifying regional variation in the developing hindbrain, however little is known of the cascade of events that regulate their expression or the process of segmentation itself. In general, *Hox* gene expression patterns are generated in two distinct phases: establishment followed by maintenance. The mechanism by which *Hox* genes become expressed at higher relative levels in specific rhombomeres is independent from the process that generates the more generalized expression patterns along the length of the neural tube. Positional values along the A-P axis appear to be conferred on the rhombomeres by differential *Hox* gene expression however it is not entirely clear how appropriate levels of expression are attained within individual rhombomeres. The observation that other genes were expressed in the hindbrain in segmentally restricted patterns overlapping with the patterns of *Hox* gene expression implied that these genes were involved in regulating *Hox* gene expression and ultimately controlling hindbrain development. Currently, there are three upstream candidate regulators of *Hox* gene expression during hindbrain development and they are the transcription factors *Krox20* and *kreisler* and the vitamin A derivative, retinoic acid.

## *KROX20* REGULATION OF *HOX* GENES

*Krox20* encodes a protein with three $C_2H_2$-type zinc fingers and was initially identified as an immediate early response gene in serum stimulated fibroblasts[62]. During early embryonic development, *Krox20* is expressed in r3 by the 8.0dpc stage, followed by r5, which occurs prior to the appearance of lineage-restricted compartments in the hindbrain (Fig. 1)[2]. *Krox20* acts as a transcription factor by binding to a specific DNA sequence and directly regulates *Hoxa2*, *Hoxb2*, *Hoxb3,* and *EphA4* through cis-acting sequences in the 5' flanking region of these genes[63,64]. *Krox20* therefore is an essential component of the upstream regulatory cascade governing hindbrain segmentation. The overlapping expression patterns of *Krox20* and *EphA4* expression suggests that transcriptional control and cell-cell signalling could be coupled together.

## *KREISLER* REGULATION OF *HOX* GENES

The *kreisler* mouse was identified due to its circling behavior, a phenotype, which is usually associated with inner ear defects[65]. *Kreisler* expression is initiated at 8.5dpc in the prospective r5 territory and is later expressed in r5 and r6 after which it is quickly downregulated in both rhombomeres (Fig. 1)[66]. The gene responsible for the *kreisler* mutation has been positionally cloned, and is a member of the *Maf* oncogenic family of b-zip (basic domain-leucine zipper) transcription factors[66]. The analysis of gene expression in *kreisler* mutant embryos strongly suggested it might play a direct role in the transcriptional regulation of genes important for hindbrain and inner ear development, particularly the *Hoxa3* and *Hoxb3* genes that are upregulated in r5 and r6. Analyses of the regulatory regions of *Hoxa3* and *Hoxb3* in mouse and chick embryos have shown that both these genes are under the direct control of the product of the *kreisler* gene[32].

## RETINOID REGULATION OF *HOX* GENE EXPRESSION

Retinoids constitute a group of vitamin A–derived signalling molecules that are involved in specifying the CNS[67,68]. Exogenous vitamin A metabolites such as retinoic acid (RA), when applied to embryos during the early stages of development, cause severe craniofacial malformations, particularly within the hindbrain and branchial arch region[67,69,70,71,72]. Cellular retinoid-binding proteins (*CRBPI* and *II*, *CRABP I* and *II*) are thought to control regional RA concentration and hence facilitate its function[73]. *CRBP* expression is confined primarily to the floor plate, where it binds retinol taken up from the blood and *CRABPI* is expressed in the mouse hindbrain in r4-6 and at lower levels in r2 at 9.5dpc[74,75]. *CRABPII* is also expressed at high levels in the hindbrain and its expression extends posteriorly along the length of the neural tube (Fig. 1)[76,77]. *CRABPs* therefore may be responsible for sequestering RA and thus limiting the amount of RA available to the nuclear RA receptors (*RAR*s and *RXR*s) that recognize particular sequence motifs within target genes called retinoic acid response elements (RAREs)[78,79]. There are three genes in each of the *RAR* and *RXR* families (a, b, and g). *RARa* is expressed up to the r3/4 border while *RARb* is expressed in the neural tube to an anterior limit coinciding with the r6/7 border[80,81]. *RXRa* and *RXRb* are expressed fairly uniformly throughout the entire hindbrain neuroepithelium (Fig. 1)[82,83]. Not all the family members however are expressed in the hindbrain

RA is the strongest candidate as an overall mediator of nested *Hox* gene expression. Excess RA causes both an anterior shift in *Hox* gene expression and an anterior to posterior transformation of regional fate[50,67,71,72,84,85,86,87,88,89,90,91,92]. Conversely, the suppression of RA signalling by the expression of dominant-negative retinoid receptors, results in anteriorization[93,94,95,96]. *Hox* genes contain the molecular machinery for responding directly to retinoid signalling[97,98,99,100,101,102,103]. The group 1 paralogs *Hoxa1* and *Hoxb1* contain 3' cis-regulatory RARE as do the group 4 paralogs *Hoxa4*, *Hoxb4* and *Hoxd4*. *Hoxa1* and *Hoxb1* are among the first *Hox* genes to be activated during development and their expression is initiated during gastrulation in response to RA, first in the newly formed mesoderm, and then in the overlying ectoderm up to a sharp anterior limit at the presumptive rhombomere 3/4 hindbrain border. As *Hoxa1* expression subsides, retinoids make a second input into *Hoxb1* regulation by refining its domain of expression to a single rhombomere segment (r4). This second input involves a negative pathway that is employed to abolish expression in the neighboring odd rhombomeres r3 and r5[99,100]. The site for repression is located within the 5' flanking region of the *Hoxb1* gene and includes another RARE, point mutations in which permit expression to spread into the neighboring rhombomeres. The application of exogenous retinoids at 7.5dpc results in the ectopic activation of *Hoxa1*, *Hoxb1,* and *Hoxa2* in more anterior tissues which is consistent with RAs perceived role as having a posteriorizing effect on the CNS. The group 4 paralogs *Hoxa4*, *Hoxb4,* and *Hoxd4* genes are also capable of responding to RA but only when administered between 8.5–9.5dpc[61,92,104]. Therefore retinoids (or more specifically RA acting via its receptor molecules [*RAR*s and *RXR*s]) are crucial in the establishment phase of *Hox* gene expression and the specification of segmental identity in the hindbrain.

## *HOX* GENE AUTO AND CROSS-REGULATION

A unique property of the group 1 paralogs is displayed by the *Hoxb1* gene which after its establishment phase, maintains high levels of expression in r4[52,54]. The continued expression or maintenance phase of *Hoxb1* in r4 does not involve RA but is achieved through a conserved autoregulatory loop involving *Hoxb1* together with an unspecified *Pbx* family protein[105]. Despite the similarities in expression and regulation between various *Hox* gene paralogs, there is frequently variation in their relative levels within specific segments. For example, of the group 2 homologs simultaneously expressed in r4, *Hoxb2* is upregulated, but in contrast, *Hoxa2* is not. This type of differential expression suggests that in even-numbered rhombomeres these two genes have distinct modes of regulation. Consequently it has been shown *in vitro* that *Hoxb1* is able to bind to *Hoxb2* in a cross-regulatory interaction[57]. A great deal of our understanding of *Hox* cross-regulation has come from analyses of the cis-regulatory regions of the group 4 paralogs, *Hoxb4* and *Hoxd4*, which have domains of expression that partially overlap and map to the r6/7 junction[106]. Similar to *Hoxb1*, *Hoxb4,* and *Hoxd4* are also capable of auto-regulation. These studies highlight the importance of auto-regulatory and cross-regulatory mechanisms for the functional maintenance of *Hox* gene expression and specification of rhombomere identity during vertebrate hindbrain development.

## *HOX* GENE SPECIFICATION OF SEGMENTAL IDENTITY AND MUTATIONAL ANALYSES

By analogy to their *Drosophila* counterparts, it was predicted that *Hox* genes would play essential roles in regulating the identity of individual hindbrain segments[4,51,52] and this has now been confirmed through null mutation and ectopic expression analyses for most of the 3' members of the *Hox* gene family. *Hoxa1* null mutants die at birth from anoxia and exhibit marked defects in the inner ear and in specific cranial nerve components[24]. The embryonic phenotype is characterized by a reduction in the size of r4 and the partial deletion of r5 as revealed by the diminished expression of *Hoxb1* in r4, and of *Krox20* and *Fgf3* in r5, and this may account for the absence of facial nerve and abducens nerve motor neurons. A second independently produced *Hoxa1* null mutant exhibits a slightly different phenotype, in which there is a complete absence of rhombomere segmentation, including the first three rhombomeres where *Hoxa1* is never expressed[25]. Nevertheless, the null mutant phenotypes indicate that the *Hoxa1* gene product is necessary for the proper segmentation of r4-6. In addition the activity of *Hoxa1* appears to be critical for the specification of rhombomeric identity because ectopic expression of this gene in r2 results in the adoption of gene expression typical of r4[107,108]. *Hoxa1* is therefore critical to both hindbrain segmentation and specification.

*Hoxb1* mutants exhibit changes in segmental identity; however, in contrast to the *Hoxa1* mutants they show no segmentation defects[30,31]. Molecular analyses indicate that the patterning of r4 is initiated properly but not maintained in *Hoxb1* mutant embryos. Rhombomere 4 specific markers such as *Wnt8c* and *CRABP1* fail to be upregulated and genetic markers of r2 are ectopically expressed in r4 suggesting that the identity of r4 has been transformed[31]. Consequently, the facial branchiomotor neurons (FBM) and contralateral vestibular acoustic efferent (CVA) neurons, which are specific to r4 are incorrectly specified. Both sets of neurons fail to migrate into the correct position leading to a loss of the facial motor nerve. These results demonstrate that as part of its role is in maintaining rhombomere identity, *Hoxb1* is involved in controlling migratory properties of motor neurons in the hindbrain. Ectopic expression of *Hoxb1* in r2 of chick embryos transforms the character of this segment into that of rhombomere 4[109]. These gain of function experiments suggest that *Hoxb1* is involved in specifying individual segmental identity.

The phenotypes of individual *Hoxa1* and *Hoxb1* loss of function (null) mutations suggest that these genes play distinct roles in hindbrain development. However, *Hoxa1/Hoxb* double mutants exhibit a surprising range of phenotypes that are absent from the individual mutants, indicating that establishment of r4 identity and patterning of the VII-XI cranial nerves requires extensive synergy between *Hoxa1* and *Hoxb1*[110,111]. In the absence of both genes, a territory appears in the region of r4, however *EphA2 (*which is one of the earliest markers of r4) fails to be activated. Not only does *EphA2* lie downstream of *Hoxa1* and *Hoxb1* in the genetic cascade that regulates hindbrain segmentation and specification, but it suggests that in the double mutants there is a failure to initiate rather than maintain the specification of r4 identity. The double mutants also revealed that *Hoxa1* and *Hoxb1* work synergistically to initiate the r4 restricted expression domain of *Hoxb1*. *Hoxa1* seems to play a role during the establishment phase of *Hox* gene expression by activating the r4 enhancer of *Hoxb1.* This is achieved through 3' RARE pararegulatory interactions. *Hoxa1* however, is unable to participate in the long-term maintenance of *Hoxb1* expression because it is not expressed at these later stages. Therefore in concert with RA, pararegulatory interactions between *Hoxa1* and *Hoxb1* initiates *Hoxb1* expression in r4, after which time, the *Hoxb1* autoregulatory mechanism kicks in to ensure that high *Hoxb1* expression levels are maintained. *Hoxb1* also functions in a direct cross-regulatory manner to upregulate *Hoxb2* expression in r4. Therefore *Hoxb1* utilizes auto-, para-, and cross-regulatory mechanisms as part of its role in maintaining r4 identity and regulating facial motor neuron patterning.

*Hoxa2* is the only *Hox* gene to be expressed in r2 and is the most anteriorly expressed of all the *Hox* genes[112]. Null mutations in *Hoxa2* result in homeotic transformations of the second arch neural crest derived elements into first arch derivatives and subsequently perinatal lethality[113]. The hindbrain is also affected to the extent that the segmental identities of r2 and r3 are altered. *EphA4* expression is selectively abolished in r2, indicating that *Hoxa2* is required for the maintenance of *EphA4* expression in r2. Consequently, the alar territories of r2 and r3 are reduced and there is a concomitant expansion of r1. In addition, the trigeminal motor axons derived from r2 and r3 migrate caudally and exit the hindbrain from the r4 facial nerve exit point rather than their normal exit point in r2. Since *Hoxa2* is the only *Hox* gene expressed in r2, these results suggest that the identity of r2 has been transformed and that it now resembles r4. *Hoxa2* therefore not only acts as a selector gene for second arch mesenchymal neural crest cells but also plays a fundamental role in rostral hindbrain patterning by establishing the identity of r2 and by influencing the migration of r2/3 derived motor axons.

In contrast to *Hoxa2* mutants, the loss of *Hoxb2* locus does not result in abnormalities to the segmental anterio-posterior organization of the developing hindbrain[114]. Rhombomeres 3 and 5 develop normally, despite the absence of *Hoxb2* upregulation in these rhombomeres. Phenotypic changes affecting dorsoventral (D-V) patterning however were observed in the hindbrain such that r4 specific neural precursors now closely resembled those originating in r2. This suggests that a subset of ventral motor neuron precursors may be incorrect specified. In addition, development of the somatic motor component of the facial nerve is impaired in *Hoxb2* null mutants, which is reminiscent of the phenotype described in the *Hoxb1* mutants. *Hoxb2* therefore plays an important role in regulating the generation and/or fate of subsets of r4 motor neuron progenitors.

The generation of *Hoxa2/Hoxb2* double mutants revealed that synergistically these genes play an important role in coupling A-P and D-V patterning[115]. *Hoxa2* and *Hoxb2* play distinct roles in mediating hindbrain neurogenesis. *Hoxa2* regulates development in the alar and dorsal basal plates of r2 and r3 whereas *Hoxb2* is essential for motorneuron development in the ventral region of r4. In the double mutants, functional synergy between *Hoxa2* and *Hoxb2* in r3 has been suggested based on the absence of a ventral interneuron subtype, which is developmentally normal in both the single mutants. In addition there also appears to be a joint requirement for *Hoxa2* and *Hoxb2* in patterning inter-rhombomeric boundaries. Despite the fact that the normal number of hindbrain segments form in the double mutants, boundary formation between r1-4 is nonexistent. Therefore these results indicate that *Hoxa2* and *Hoxb2* differentially control alar and basal plate development within distinct rhombomeres. Perhaps more importantly this provides a link between *Hox* mediated patterning along the A-P axis and neurogenesis along the D-V axis and can account mechanistically for the generation of neurons at reproducible positions within hindbrain segments.

A targeted mutation in the *Hoxa3* gene results primarily in mesenchymal neural crest defects[116]. The *Hoxa3* null mutant mice are athymic, aparathyroid, and have throat cartilage malformations. In addition, the loss of *Hoxa3* leads to defects in the formation of the IXth cranial nerve and also fusions between the IXth and Xth nerves. Examination of the cranial nerves in *Hoxb3*-/- embryos revealed similar cranial ganglia defects but at a lower penetrance than in the *Hoxa3* mutants[117]. In contrast, the cranial nerves in the *Hoxd3*-/- embryos are completely normal[118,119]. Therefore at least two of the group 3 paralogs play essential roles in formation of the IXth cranial ganglion. Double mutants (*Hoxa3/Hoxb3*, *Hoxa3/Hoxd3,* and *Hoxb3/Hoxd3*) were subsequently generated and in *Hoxb3/Hoxd3* double mutants there was a marked increase in the penetrance of the IXth cranial ganglion defects relative to that observed in the *Hoxb3* single mutant[118,119,120]. This increase in penetrance shows that *Hoxd3* plays a synergistic role with *Hoxb3* in cranial nerve development even though the *Hoxd3* single mutant does not show a defect in these structures.

## *KROX20* NULL MUTATIONS

The necessity for coupling cell identity and cell lineage restrictions in order to generate sharply restricted and specified segmental domains during hindbrain development is highlighted in the phenotype of *Krox20* null mutants. Mice homozygous for a targeted mutation in *Krox20* die shortly after birth and exhibit fusions of the trigeminal ganglion with facial and vestibular ganglia as a consequence of the profound perturbation of hindbrain morphogenesis[26,27,121]. Although the presumptive territories of rhombomeres 3 and 5 do form during the very early stages of hindbrain development, these two rhombomeres are not maintained and their structural derivatives are subsequently eliminated. Consistent with the interactions unveiled by transgenic analyses between *Krox20* and *Hoxa2*, *Hoxb2*, *Hoxb3,* and *EphA4*, no upregulation of these three genes was observed in *Krox20* mutants. Therefore *Krox20* is a key regulator of gene expression and segmentation in the developing hindbrain.

## KREISLER *mutants*

The *kreisler* mutation is not a null allele, but a regulatory mutation affecting those enhancer elements responsible for the hindbrain domain of expression of the gene[66]. The primary defect in *kreisler* mutant embryos is a disruption of segmentation in the otic region of the hindbrain, whereby the rhombomeric borders that normally demarcate r4, 5, and 6 are absent[32,65,122]. Consequently the normal expression domains of *Fgf3* and *CRABPI* and the upregulation of *Hoxa3* in r6 are absent. Although *Krox20* expression in presumptive r3 is present, the posterior band associated with r5 is missing. Similarly, the normal expression domains of *Hoxb2*, *Hoxb3,* and *Hoxb4* in r5 are also abolished. Analysis of patterns of expression of *EphA7* and *ephrinB2* indicate that only a single segment correlating with r5 is missing. Therefore the segmentation defect associated with the *kreisler* mutant is a specific loss of r5 and although an r6 territory does form, it fails to mature[32]. Through direct regulation of *Hox* genes, *kreisler* controls segmental identity and this is further evidenced by the ectopic activation of *kreisler* in r3 which transforms the character of this territory into that of r5[123]. The role played by *kreisler* in hindbrain patterning is therefore analagous to *Krox20* because rather than being restricted to a single patterning aspect, *kreisler* is involved at several different levels including establishment and maintenance of A-P identity in addition to segment specification.

Clearly hindbrain segmentation in the form of cell lineage restrictions and compartmentalization is coupled with the acquisition of specific cellular identities. The studies described above illustrate that *Hox* genes function in many steps of hindbrain segmentation process particularly in specifying segmental identity. Consequently hindbrain segmentation and rhombomere specification plays important roles in patterning the craniofacial structures of neural crest origin.

## CRANIAL NEURAL CREST CELLS AND THE PREPROGRAMMING MODEL FOR CRANIOFACIAL DEVELOPMENT

Neural crest cells were so named because of their formation along almost the entire axis of vertebrate embryos at the crest of the closing neural folds. This region corresponds to the interface or junction between the non-neural ectoderm (presumptive epidermis or surface ectoderm) and the neural plate (neuroepithelium), a region commonly referred to as the neural plate border (Fig. 2). Neural crest cell induction requires contact-mediated interactions between the surface ectoderm and neuroepithelium and importantly, each of these tissues contributes to the neural crest cell lineage[124]. BMP signalling is thought to play critical roles in both positioning the border of the neural plate during gastrulation and then also later in inducing migration of neural crest cells[125]. Cranial neural crest cells give rise the neurons and glia of the PNS and most of the cartilage, bone, and connective tissue of the face. The cartilage and bone forming properties of cranial neural crest cells distinguishes this population from trunk neural crest cells. Hence the cranial neural crest is a pluripotent population that is vital to vertebrate head development (Fig. 2)[126,127,128,129,130,131]. It is essential to understand the mechanisms by which cranial neural crest cells are patterned since many craniofacial malformations can be attributed to defects in the formation, proliferation, migration, and/or differentiation of this cell population.

The generation of regional diversity in the vertebrate head is believed to be a consequence of patterning information provided by neural crest cells during their migration and neural fold transplantation experiments have provided much of the basis for our current understanding of neural crest and craniofacial development[132,133,134,135,136]. First arch neural crest cells give rise to Meckel's cartilage, the articular, quadrate, and squamosal bones. In contrast the second arch neural crest cells develop into the hyoid or tongue cartilage and the retroarticular process[9,136,137]. In posterior transplantations of first arch (mandibular) neural crest primordia in place of second (hyoid) or third (visceral) arch neural crest, the transplanted neural crest cells migrated into the nearest arch but therein formed ectopic duplicated proximal first arch skeletal elements[136]. Not only were these duplicated crest-derived structures inappropriate for their new location but the muscle cell types and attachments associated with the duplicated structures were also characteristic of a first arch pattern. This suggested firstly that neural crest cell fate may be preprogrammed prior to their emigration from the neural tube and secondly, that myogenic populations and other cell types receive spatial cues from the invading neural crest-derived connective tissue. The vast majority of cranial neural crest cells are derived from the hindbrain and molecular evidence supporting this scenario was provided by the observation that the same restricted domains of *Hox* gene expression in the hindbrain are emulated in the migrating neural crest cells and then later in the ganglia and branchial arches, reflecting the origins of the neural crest cells contributing to these tissues[4,51]. Under this preprogramming model, it was thought that positional information encoded by the *Hox* genes was carried passively from the hindbrain to peripheral tissues and branchial arches by the neural crest, where it was elaborated to form the characteristic head structures. Therefore it was hypothesized that the spatial organization of structures within the vertebrate head was determined by the neural crest and furthermore that the positional information imparted by the neural crest was irreversibly set before the neural crest emigrates from the neural tube.

## CRANIAL NEURAL CREST PLASTICITY AND INDEPENDENT *HOX* GENE REGULATION MODEL

The neural crest prepatterning model predicts that molecular and cellular alterations to the segmental organization of the hindbrain would have profound and detrimental consequences to craniofacial development via disruptions to the patterns of *Hox* gene expression and neural crest cell migration. The chick embryo has been the primary species for testing this model via rhombomere transplantations, rotations, and ablations owing to the ease of tissue manipulation in this species[112,138,139,140,141,142]. These analyses have yielded conflicting results regarding the degree of autonomy of *Hox* gene expression[143]. The recent application of techniques for transposing cells to the hindbrains of mouse[144] and zebrafish[145] embryos has significantly advanced our understanding of hindbrain and neural crest development. In the mouse, cells from r3, r4, or r5 were heterotopically grafted into r2. The majority of the transplanted cells remained as a cohort and maintained their *Hox* gene A-P identity. A few transplanted cells however, became separated from the primary graft and dispersed becoming intermingled with the neighboring populations. These cells consequently altered their identity in their new location by failing to maintain the *Hox* gene expression patterns characteristic of their origins, which is evidence for neuroepithelial plasticity. This implies that single or dispersed rhombomere cells respond and adapt to their new surrounding environments by altering their gene expression patterns because they lack the neighboring signals necessary to reinforce or maintain their original identity[144]. Similarly, single rhombomere cell transplantations in zebrafish provide further evidence for neural crest plasticity and the influence of cell community effects[145]. A complete switch in *Hox* gene expression patterns and ultimately cell fates occurred when single cells from r2 and r6 were reciprocally transplanted within the zebrafish hindbrain. This contrasts with the higher degrees of cell autonomy observed in chick embryo manipulations. It is important to note however that, in analyses performed in avian embryos, pairs of rhombomeres but generally much larger regions of the neural tube were being manipulated. It is not unreasonable to expect that cell community effects present in such large tissue grafts could account for the cell autonomy observed in these avian studies compared to the plasticity observed in mouse and zebrafish experiments where subrhombomeric or single cell populations were transplanted, respectively[143]. The degree of plasticity or autonomy is dependent upon not only the size of the graft but also the time at which the manipulations are carried out. During the later stages of hindbrain development, when morphological boundaries are established, rhombomere cells are much more likely to be irreversibly committed and maintain their *Hox* gene expression characteristics when transplanted to ectopic locations. Therefore one could argue that over time cells within the neural tube progressively lose responsiveness to the environmental signals that specify their segmental identities. Together these chick, mouse and fish studies highlight that cell-community effects and their associated signals are important for maintaining the axial identity of individual cells within the hindbrain.

These grafting experiments also revealed the absence of preprogramming in the character or fate of cranial neural crest cells. In heterotopic transpositions of cells within the mouse and zebrafish hindbrains, graft-derived neural crest cells migrated into the nearest branchial arch without any evidence of pathfinding or rerouting to their original axial level. Plasticity in *Hox* gene expression in mouse neural crest cells was evident by the complete down regulation of *Hoxb1*, *Hoxb2*, and *Hoxa2* in these cells[144]. In zebrafish, experimental embryos raised to larval stages revealed that the transplanted cells differentiated and contributed to pharyngeal cartilages appropriate to their new A-P location[145]. Therefore these results show that the genetic and cellular A-P character of cranial neural crest cells are neither fixed nor passively transferred from the hindbrain to the branchial arches.

The neural plasticity described above correlates with molecular analyses that have identified distinct regulatory elements controlling *Hox* gene expression in different tissues such as the hindbrain and neural crest. *Hoxa2* is expressed in the hindbrain anteriorly up to the r1/2 boundary and in cranial neural crest cells that migrate into the second branchial arch[112]. Transgenic regulatory analyses of *Hoxa2* have revealed that multiple cis-acting elements are required independently for hindbrain and neural crest specific activity[55,146,147]. In r3 and r5, *Hoxa2* expression is directly regulated by the transcription factor *Krox20*[55]. In contrast, *Hoxa2* expression in second branchial arch neural crest cells is tightly controlled by a number of elements, one of which binds to *AP-2* family members. Mutation or deletion of this site in the *Hoxa2* enhancer abrogates expression in cranial neural crest cells but not in the hindbrain. These findings clearly demonstrate that at the molecular level, *Hoxa2* is independently regulated in rhombomeres and neural crest cells and this provides a mechanism for how neural crest cells can respond to the environment through which they migrate independently of the neural tube.

These experiments have revealed a surprising degree of plasticity in cranial neural crest cells, which is inconsistent with the prepatterning model. Since transposed neural crest can be reprogrammed it appears that crest cells rely on distinct cues in the branchial arch environments through which they migrate to elaborate their proper regional identity. Furthermore, the size of

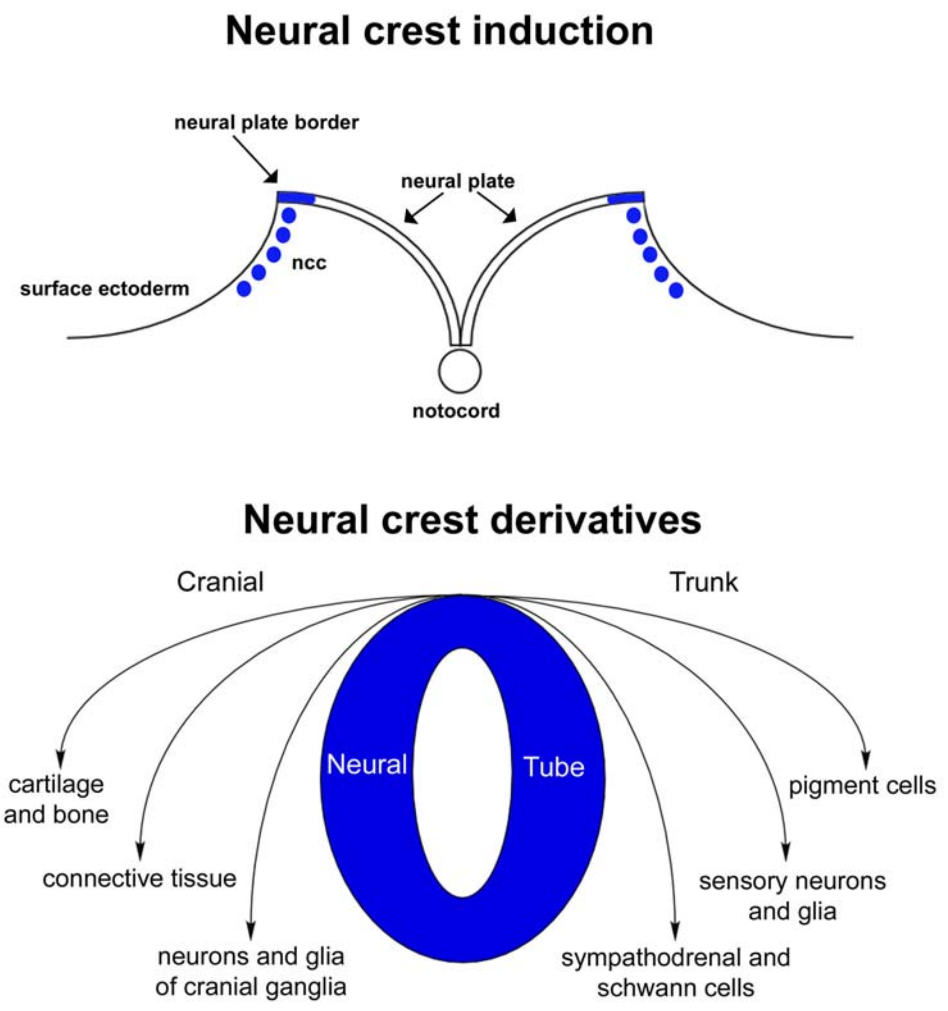

**FIGURE 2**. Neural crest cell induction and differentiation. Neural crest cells (ncc; blue circles) are induced at the neural plate border, which demarcates the junction between the neural plate and surface ectoderm. Both the neuroepithelium and the surface ectoderm gives rise to neural crest cells and contact between these tissues is essential for neural crest induction. In the mouse as depicted here, this occurs prior to closure of the neural tube. In contrast, in avian species the neural tube is closed prior to the emigration of neural crest cells. After migrating along stereotypic routes, the cranial neural crest cells differentiate into cartilage, bone, connective tissue as well as neurons and glia of the PNS. In contrast, trunk neural crest cells do not give rise to cartilage or bone rather they differentiate predominantly into the characteristic cells that constitute the trunk PNS.

the cell community is functionally important, indicating that a far more complex balance of genetic and cellular interactions are involved in hindbrain and neural crest patterning than was previously thought. The experiments described above therefore provide the basis for a new model, the neural crest plasticity and independent *Hox* gene regulation model which has been proposed as an alternative mechanism for governing neural crest and craniofacial development[143,144,148].

## EVOLUTION AND PATTERNING OF NEURAL CREST CELLS

The classic neural crest preprogramming model implies that branchial arch formation and patterning is dependent upon the neural crest. In contrast, the neural crest plasticity and independent gene regulation model described above implies that branchial arch patterning arises due to interactions between the arch components and the neural crest. This raises the question of what happens to the formation and patterning of the branchial arches in the absence of contributing neural crest cells. This issue has been investigated in chick embryos through rhombomere ablations[149] and also mouse embryos by genetic manipulation of *Hoxa1* and *Hoxb1*, which are required for the generation of neural crest cells in r4[33]. In both experimental situations, despite the absence of any neural crest cell contribution, the branchial arches develop normally and are properly regionalized. The expression patterns of *Bmp7* in the posterior endoderm, *Fgf8* in the anterior surface ectoderm, *Pax1* in the pharyngeal pouch endoderm and *Shh* in the endoderm of the branchial arches were all normal and unchanged compared to wild type embryos. In addition there was no evidence for excessive cell death or loss of proliferation in the arch epithelium, which suggests that the neural crest cells are not the source of any indispensable branchial arch mitogenic or survival signals[33]. These results clearly demonstrate that the branchial arches are not dependent upon the neural crest for their formation, nor for their anterior-posterior and proximo-distal regionalization and hence this provides additional support for the neural crest plasticity and independent gene regulation model. This is also consistent with the evolutionary history of the branchial arches and neural crest cells. Pharyngeal segmentation is characteristic of the phylum chordata whereas neural crest cells are exclusively a craniate (vertebrates plus hagfish) characteristic[150], which implies that branchial arch segmentation occurred prior to the evolutionary origin of cranial neural crest cells. Hence it is not surprising that the branchial arches do not depend upon the cranial neural crest for their initial formation and regional specification. Additional support for this idea comes from the observation of regionalized domains of *Pax* gene expression in Amphioxus (the nearest extant vertebrate relative), which is indicative of pharyngeal segmentation. Amphioxus lack neural crest cells and therefore the mechanism for generating pharyngeal pouches clearly predates the evolution of the vertebrate head[151].

## PATTERNING ROLES FOR THE MESODERM, ENDODERM, AND ECTODERM IN BRANCHIAL ARCH DEVELOPMENT

A crucial issue in craniofacial development is understanding the mechanisms that regulate the size and shapes of the characteristic facial skeletal structures and that branchial arch segmentation constitutes one of the first visible steps in this patterning process. Since normal branchial arch formation and patterning can occur independently of a contribution from the neural crest, this implies that the branchial arches may rely on either the endoderm, paraxial mesoderm, and/or the surface ectoderm tissues for their patterning information.

### The Mesoderm

Fate mapping studies have shown that mesoderm and neural crest cells derived from the same axial level contribute to the same branchial arch during embryonic development (Fig. 3)[16,130,152,153]. The cranial mesoderm predominantly gives rise to the myogenic cores of each branchial arch, which are enveloped by migrating neural crest cells[16,130,131,153,154]. Previously, the cranial mesoderm was not thought to play a patterning role during craniofacial development[136], however, it has now been shown that the cranial mesoderm provides maintenance signals for regulating the identity of second branchial arch neural crest cells[144] and that it may also play a role in patterning the pathways of neural crest cell migration (Fig. 3)[23]. When second arch neural crest cells are transplanted into the first arch, they downregulate their expression of *Hoxb1*. In contrast, if second arch neural crest cells are transplanted anteriorly in combination with second arch mesoderm, then *Hoxb1* expression is maintained in the grafted neural crest cells. The cranial mesoderm therefore provides maintenance signals that elaborate the programme of *Hox* expression, but the cranial mesoderm does not appear to initiate *Hox* gene expression in neural crest cells[144]. It is important to note however that *Hoxb1* is expressed in the cranial mesoderm at 7.5dpc prior to its induction in the neuroepithelium suggesting that perhaps the mesoderm is patterning neuroepithelial tissues at much earlier stages of development. The effects of the mesoderm are consistent with the fact that the fate of the cranial mesoderm is primarily myogenic and the musculature is inextricably linked to neural crest derived skeletal and connective tissue patterning. Therefore one of the roles of the cranial mesoderm maybe in maintaining an A-P register between these different primordial tissues, which is essential for subsequent craniofacial morphogenesis[16].

## The Ectoderm

Similar to the neuroepithelium, it has been suggested that the ectoderm is regionalized into territories called ectomeres, which contribute to specific regions of the branchial arches (Fig. 3)[155]. Currently, there is no evidence to support the idea that each ectomere represents a functional developmental unit. In contrast, however, there is evidence suggesting that the surface ectoderm plays a major role in the induction of odontogenesis during branchial arch development[156]. The oral ectoderm of the first branchial arch directly regulates the patterning of the underlying neural crest mesenchyme into teeth and the ability to respond to these instructive or inducing signals is not confined to first arch neural crest cells[157]. *Fgf8*, which is expressed in the anterior surface ectoderm of the first arch, is essential for determining the polarity of the branchial arch and ectopic applications of FGF8 cause shifts in gene expression domains as well as repatterning of the craniofacial primordia[158]. Not surprisingly then, in *Fgf8* null mutant mice, the branchial arches are severely abnormal[159]. *Bmp4*, which is expressed in the ventral region of the first branchial arch ectoderm, appears to restrict the expression domain of *Fgf8* and consequently ectopic applications of BMP4 consistently reduce the size of the mandibular arch. Hence the surface ectoderm plays important roles in patterning the branchial arch derivatives particularly through the BMP4 and FGF8 signalling mechanisms (Fig. 3).

## The Endoderm

The neurogenic placodes (dorsolateral and epibranchial) form in characteristic positions in all vertebrates suggesting that conserved localized inductive interactions underlie their formation[160]. The epibranchial placodes develop near the branchial clefts in close proximity to the cranial neural crest and the pharyngeal endoderm. Analyses of the nature of the signals, which underlie epibranchial placode formation, have found that the epibranchial placodes do not require cranial neural crest cells for their induction[161]. Rather, it is the pharyngeal endoderm that is the source of the BMP7 inducing signal. It has been suggested that neural crest cells will differentiate into cartilage only in the presence of pharyngeal endoderm and in amphibians, the endoderm has been shown to be responsible for promoting the formation of branchial arch components by directing neural crest cells towards a chondrogenic fate[162]. For example, in the zebrafish mutant *van gogh* (vgo), hindbrain segmentation occurs normally, but the endodermal gill slits do not form and the distinct streams of neural crest cells exiting from the hindbrain are fused.

Consequently the individual skeletal elements of the viscerocranium do not form[163]. Therefore the endoderm plays a major role in establishing and patterning the branchial arches (Fig. 3). Recently the avian neural endoderm was tested in transplantation and ablation studies for its capacity to specify the facial skeleton[164]. The experiments suggested that the endoderm instructs neural crest cells as to the size, shape, and position of all the skeletal elements whether they are cartilaginous or membranous bones. In addition bone orientation was shown to be influenced by the position of the endoderm relative to the embryonic axes. The nature of the signals arising from the endoderm are so far unknown and it is not clear whether the effects of the endoderm manipulations are direct and intrinsic to the tissue itself or whether the effects of the manipulations indirectly alter local signaling centers or levels of FGF, Shh, or BMP which have been shown in other analyses to regulate the development of the characteristic craniofacial structures[159,165,166,167]. What is clear is that segmental characteristics of the endodermal pharyngeal pouches develop independently of a contribution of neural crest cells (Fig. 3)[149].

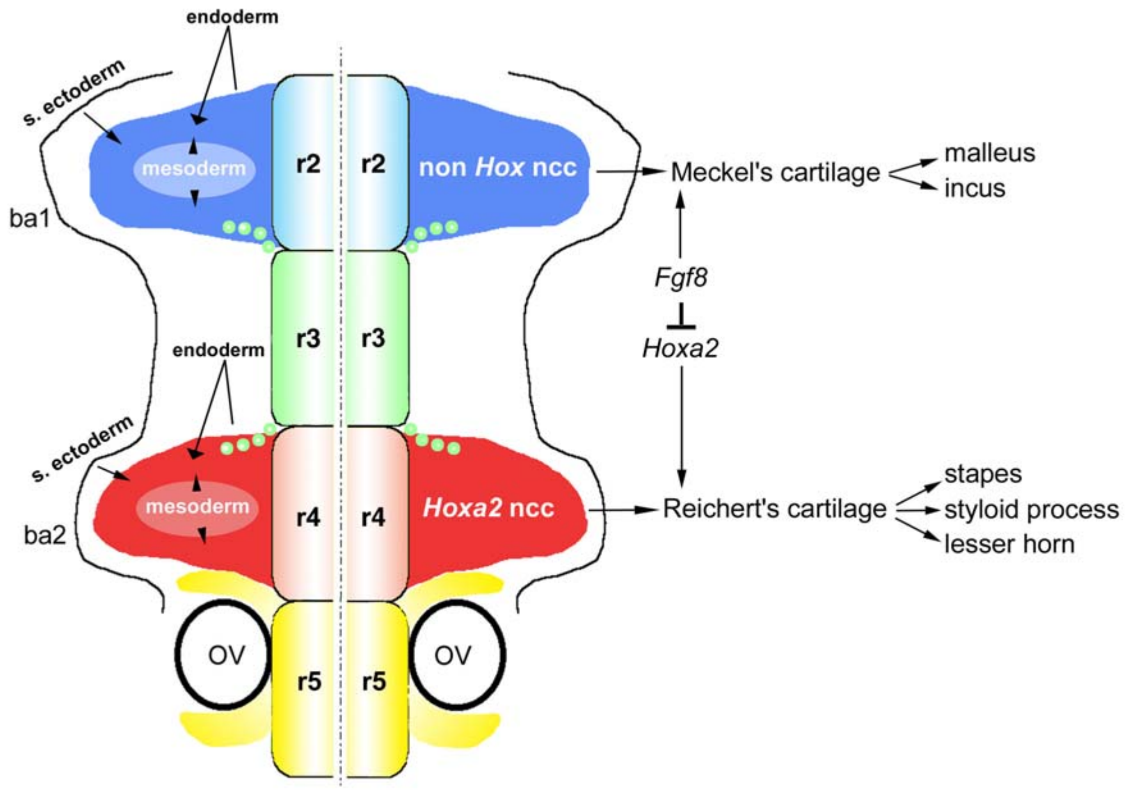

**FIGURE 3**. The influence of mesoderm, ectoderm, and endoderm on the axial identity of neural crest cells and the role of *Hoxa2* in second branchial arch development. The head mesoderm (red and blue ovals) plays a role in maintaining the proper domains of *Hox* gene expression in migrating neural crest cells (ncc), whereas the ectoderm and endoderm play roles in possibly influencing the pathways of neural crest cell migration in addition to patterning the branchial arches. The skeletal derivatives arising from the first (ba1) and second (ba2) branchial arches are listed. *Hoxa2* is expressed in neural crest cells migrating into the second arch but not the first arch acts and as a selector gene that imposes the unique identity of second arch structures. In the absence of *Hoxa2*, second arch derivatives are transformed into a first arch fate and similarly ectopic expression of *Hoxa2* transforms first arch structures into a second arch identity. FGF8 has the capacity to repress *Hoxa2* in second branchial arch neural crest cells, transforming the second arch into a first arch identity. OV, otic vesicle.

## RESOLVING THE ISSUES OF NEURAL CREST PLASTICITY VS. PREPATTERNING AND SKELETAL DUPLICATIONS

The analyses detailed above provide a wealth of evidence supporting the idea that cranial neural crest cells are not preprogrammed, but are in fact plastic and that they differentiate in response to signals from the environment through which they migrate. How then do we reconcile these findings with the skeletal duplications observed in posterior transplantations of presumptive first arch neural crest[136]? Two things are often ignored from this classic study. Firstly, in addition to forming duplicated first arch structures, the transplanted neural crest also contributed to the development of normal second arch skeletal elements including the paraglossals and basihyoid, which make up part of the tongue skeleton. This actually provides evidence for neural crest plasticity. Secondly, when presumptive frontonasal neural crest was grafted posteriorly in place of second arch neural crest, duplicated first arch skeletal elements (quadrate and proximal region of Meckel's cartilage) also developed, providing further evidence in support of neural crest plasticity[136]. It's important to note here that the same duplicated skeletal structures were formed regardless of the origin of the grafted neural crest cells.

What's intriguing about the skeletal duplications is that they phenocopy the *Hoxa2* null mutant mouse in which the second arch is transformed into a first arch identity and one can speculate then that perhaps the landmark chick embryo manipulations were in effect creating a conditional knockout of *Hoxa2* in the second branchial arch. Recently, the isthmus was shown to inhibit *Hoxa2* expression in rhombomere 1 via an FGF8 mediated signalling mechanism[168], and therefore one plausible explanation which could link the results of the two transplantations together is the possible inclusion of the isthmus in both the first arch and frontonasal neural crest grafts. This has now been tested directly via posterior transplantations of the isthmus in place of r4 that inhibits the expression of *Hoxa2* in second branchial arch neural crest cells[169]. In the absence of *Hoxa2* expression, as expected these grafted chick embryos develop duplicated first arch skeletal structures including the quadrate and proximal portion of Meckel's cartilage similar to the classic transplantations. FGF8 soaked beads can only transiently block *Hoxa2* expression in second branchial arch neural crest cells indicating that FGF8 cannot recapitulate the entire effects of the isthmus and that other genes/factors must be involved.

Therefore the possible inclusion of the isthmus in the transpositions and the ability of FGF8 to suppress *Hoxa2* in second arch neural crest cells can account mechanistically for the development of duplicated first arch structures in ectopic posterior locations. Hence rather than providing evidence for neural crest preprogramming, Noden's 1983 experiments highlight the effects of local signaling centers like the isthmus in A-P patterning and regulation of *Hox* gene expression by FGFs[136]. Together with recent evidence from mouse, chick, and zebrafish transplantation studies, this argues as a general principle that cranial neural crest cells are not prespecified or irreversibly committed prior to their emigration from the neural tube. Rather, that neural crest patterning is based on plasticity and the ability of neural crest cells to respond to environmental signals and interactions with the tissues through which they migrate[144,148].

Recently inroads have been made into the mechanisms by which *Hoxa2* influences the morphogenesis of second arch elements[170]. During normal development, *Hoxa2* is widely expressed in the second arch mesenchyme, but it is excluded from the chondrogenic condensations in the core of the arches (Fig. 3). In the absence of *Hoxa2*, ectopic chondrogenesis occurs indicating that *Hoxa2* acts upstream very early in the chondrogenic pathway. In addition, *Cbaf1,* which is a marker of osteogenic differentiation, is upregulated in the second branchial arches of *Hoxa2* mutant embryos suggesting that the prevention of *Cbfa1* induction might mediate *Hoxa2* inhibition of dermal (intramembranous) bone formation during second arch development[170]. Therefore *Hoxa2* is essential for proper patterning of structures derived form the second branchial arch neural crest cells as it inhibits membranous ossification. Ectopic expression of *Hoxa2* in the first arch of chick and *Xenopus* embryos results in the suppression of bone formation in the first arch and transformation of the first arch into second arch structures[171,172]. In the absence of *Hoxa2* the second arch is transformed into a first arch identity and together these results indicate that *Hoxa2* acts as a positive selector gene in specifying second branchial arch development and identity (Fig. 3).

## EVOLUTIONARY IMPORTANCE AND CONCLUSIONS

Craniofacial evolution is considered fundamental to the origin of vertebrates and in evolutionary terms the vertebrate head is a relatively new structure[173]. This review has detailed the multiple levels of regulation and the diverse tissue interactions that are involved in generating the characteristic craniofacial features. Although the hindbrain exerts a profound influence in establishing the foundations of vertebrate head development, a rigid prepatterning model in which the programs for head morphogenesis were set in the hindbrain would however offer restricted opportunities for diversifying head structures[143,144,148]. In contrast the neural crest plasticity and independent gene regulation model could provide the flexibility and adaptability that facilitates diversity and we can speculate that it might be one reason for the successful radiation of vertebrates into new environments. This is because neural crest plasticity and independent gene regulation offers the potential for generating substantially distinct cranial phenotypes by minor changes of the primordial pattern. Future studies therefore will be focused on elucidating the mechanisms governing the formation and patterning of neural crest cells as they are critical to understanding craniofacial evolution and the origins of vertebrates.
